# Anterior quadratus lumborum block for ambulatory laparoscopic cholecystectomy: a randomized controlled trial

**DOI:** 10.3325/cmj.2021.62.137

**Published:** 2021-04

**Authors:** Jan Sverre Vamnes, Marie Sørenstua, Knut Inge Solbakk, Birgitte Sterud, Ann-Chatrin Leonardsen

**Affiliations:** Department of Anesthesiology, Østfold Hospital Trust, Moss, Norway

## Abstract

**Aim:**

To explore the effects of an anterior quadratus lumborum block (QLB) on opioid consumption, pain, nausea, and vomiting (PONV) after ambulatory laparoscopic cholecystectomy.

**Methods:**

This randomized controlled study recruited 70 patients scheduled for ambulatory laparoscopic cholecystectomy from January 2018 to March 2019. The participants were randomly allocated to one of the following groups: 1) anterior QLB (n = 25) with preoperative ropivacaine 3.75 mg/mL, 20 mL bilaterally; 2) placebo QLB (n = 22) with preoperative isotonic saline, 20 mL bilaterally; and 3) controls (n = 23) given only standard intravenous and oral analgesia. The primary endpoint was opioid analgesic consumption. The secondary endpoints were pain (numeric rating scale 0-10) and PONV (scale 0-3, where 0 = no PONV and 3 = severe PONV). Assessments were made up to 48 hours postoperatively.

**Results:**

The groups did not significantly differ in opioids consumption and reported pain at 1, 2, 24, and 48 hours postoperatively. PONV in the QLB group was lower than in the placebo and control groups.

**Conclusion:**

Preoperative anterior QLB for laparoscopic cholecystectomy did not affect postoperative opioid requirements and pain. However, anterior QLB may decrease PONV.

**Clinical Trial number:**

NCT03437187; January 22, 2018.

Inadequate post-operative pain control may lead to adverse outcomes, such as prolonged hospitalization, higher incidence of re-operation, re-admission, and higher treatment costs (1-3). After laparoscopic cholecystectomy, 17%-41% of patients have been shown to suffer insufficient pain relief (4). However, this may be counteracted by an effective postoperative analgesic treatment.

The evolution of nerve stimulation techniques and ultrasound-guided regional anesthesia have greatly enhanced the success and quality of peripheral nerve blocks (5,6). In the area of thoraco-abdominal surgery, a variety of nerve blocks has been introduced. A detailed injection technique of the earliest variants of the quadratus lumborum block (QLB) was described in 2013 (7). All thoracolumbar blocks are injected close to the thoracolumbar fascia (TLF) (8,9), but the target area is different, as well as the spread of local anesthetics and clinical effects (10). The QLB is described in different anatomical locations – the lateral, posterior, and anterior location (11,12). In the lateral QLB, the anatomical target is any point lateral to the QL muscle, while in the posterior QLB, the target is a point between the muscle and the middle layer of the TLF. The anterior QLB anatomical target is between the QL and the psoas major muscle, in the anterior layer of the TLF (13), from where local anesthetics have been shown to spread into the thoracic paravertebral space (14).

QLB provides early and rapid pain relief and allows early ambulation in certain patient populations. Multiple case studies also confirmed the QLB to be a rescue block after different surgical procedures (13,15,16). Complications associated with the performance of abdominal wall blocks are fortunately very rare (17). However, studies on the effect of anterior QLB on postoperative opioid consumption are scarce.

The primary aim of this study was to evaluate the effect of using the anterior QLB in ambulatory, laparoscopic cholecystectomy, as measured by opioid consumption (primary outcome), experienced pain, and postoperative nausea and vomiting (PONV) (secondary outcomes). The anterior approach to the QLB was chosen since studies have shown that the spread of local anesthesia to the thoracic paravertebral space reduces visceral pain (13,18-22), and that the block has a longer duration than other QLB approaches (23).

## Patients and methods

### Study design, setting, and participants

**Figure 1:**
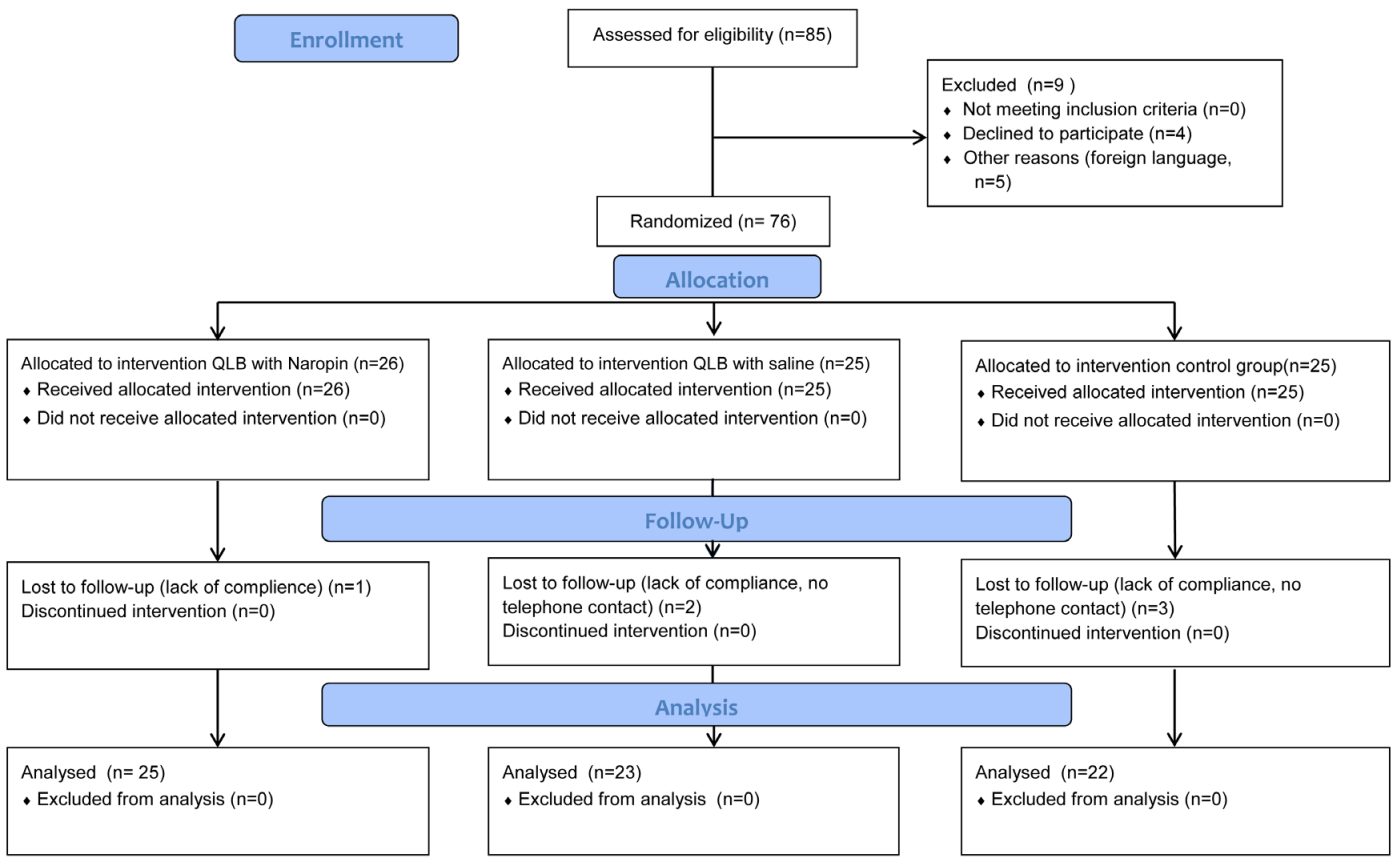
Flowchart of the inclusion process. Abbreviations: QLB – quadratus lumborum block.

This randomized, controlled, single-blinded study was conducted in a hospital trust in southeastern Norway, serving approximately 300 000 inhabitants. The hospital performs approximately 17 000 surgical procedures every year. The inclusion criteria were age >18 years, American Society of Anesthesiologists Physical Status I and II, body mass index between 20 and 35 kg/m^2^, and being scheduled for ambulatory laparoscopic cholecystectomy. The exclusion criteria were allergy to local anesthetics, chronic pain requiring opioid analgesics, atrioventricular block II, treatment with class III antiarrhytmics, severe renal and/or hepatic disease, coagulation disorders, or infection injection site (Figure 1).

The patients were examined by an anesthesiologist before surgery. Eligible patients received written and oral information before inclusion, and signed an informed consent. The enrollment lasted from January 2018 to March 2019. The study was approved by the Norwegian Regional Committee for Medical and Health Research Ethics (2017/1894).

### Randomization

Random allocation was performed with block randomization (random block sizes) using the online tool “*randomization.com*.” We used a sequentially numbered opaque sealed envelope system. Seventy-six patients scheduled for ambulatory laparoscopic cholecystectomy were randomly allocated to one of the following groups: 1) group QLB; preoperative: ropivacaine 3.75 mg/mL, 20 mL bilaterally; 2) placebo group; preoperative: isotonic saline, 20 mL bilaterally; and 3) control group: intravenous and oral analgesics only.

The patients, the nurses in the postoperative anesthesia care unit (PACU), and the researcher who conducted the postoperative telephone interviews with the patients at home, were blinded to the group assignments, except randomization to the control group.

The sample size was calculated based on clinical evaluation of opioid consumption at 1 hour postoperatively, and a 20% reduction was assumed significant (1-beta set to 0.80). The chosen critical alpha level was set at 0.05. The calculations showed that the required sample size was 69 patients (23 in each group). When adjusting for potential drop-outs and patients lost to follow-up, we determined that the adequate sample size would be 75 patients.

### The quadratus lumborum block

**Figure 2:**
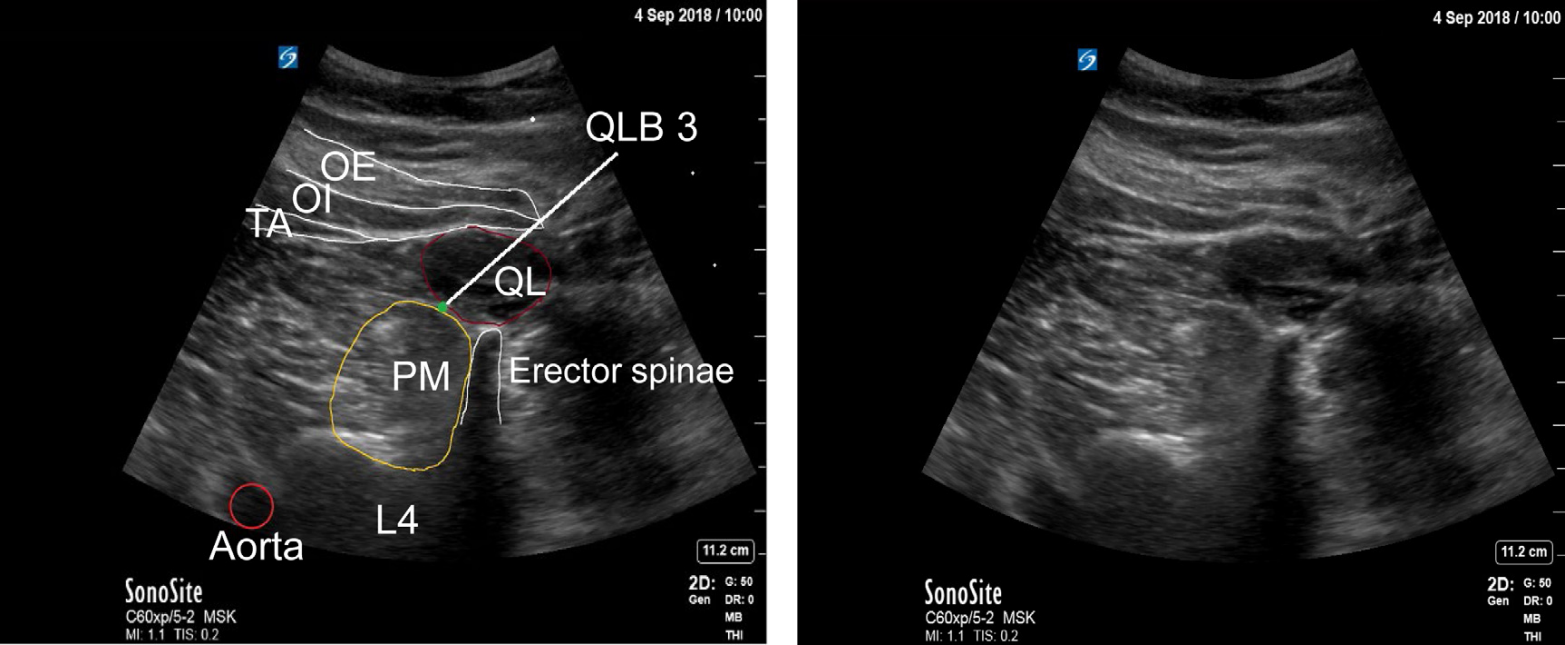
The nerve block placement in the anterior quadratus lumborum block (QLB). Abbreviations: TA – transversus abdominis; OI – oblique internal; OE – oblique external; PM – psoas major.

The bilateral QLBs were performed before the induction of general anesthesia by two staff anesthesiologists (KIS and BS) experienced in the ultrasound-guided QLB technique, who were aware of the type of injected solution. The patients were transferred to the PACU before the operation and underwent standard monitoring with a three-lead EKG, pulse oximetry, and non- invasive blood pressure monitoring. They were then positioned in the lateral decubitus position, and the transmuscular QLB was performed bilaterally using an ultrasound unit (X-Porte, FujiFilm, Sonosite, Bothell, WA, USA) with either a curvilinear transducer (5-2 MHz C60XP) or a curved transducer (8-3 MHZ C35xp). The skin was prepared with two applications of 5% chlorhexidine-added phenol red, and the probe was covered with sterile covering. The probe was positioned superior to the iliac crest, in the transverse orientation, at the posterior axillary line. The Shamrock sign (24) was identified at the L4 level, and a 20 G, 100-mm needle (Stimuplex Ultra 360, Braun, Kronberg, Germany) was advanced from posterior to anterior, through the QL muscle, until the needle tip was visualized in the interfascial plane between the QL muscle and psoas muscle. As the correct needle placement was confirmed with 2-3 mL of saline, 20 mL of 0.375% ropivacaine was injected bilaterally in the intervention group, and 20 mL of 0.9% normal saline in the control group. Four patients weighing <65 kg received a dose reduction to 20 mL 0.3% bilaterally so as to not exceed the toxic limits (3 mg/kg for ropivacaine) (Figure 2).

### Procedure

All participants followed a standardized perioperative procedure: premedication with paracetamol 2 g per os (po) and diclofenac 100 mg po at least 1 hour before surgery; general anesthesia using a target-controlled infusion of propofol and remifentanil; ondansetron 4 mg and dexamethasone 8 mg iv preoperatively; endotracheal intubation performed after topical spray with lidocaine 50 mg. No muscle relaxant was used throughout the procedure. The surgical approach involved applying two 5-mm ports and two 12-mm ports umbilically and supra-umbilically. The pneumoperitoneum was established and maintained at 12 mm Hg. All ports were closed with resorbable sutures. Intravenous (iv) oxycodone 5 mg was given when the laparoscopic instruments were removed from the patient. Subcutaneous port hole infiltration at the end of surgery was performed with ropivacaine 2 mg/mL, 20 mL.

Postoperatively, oral paracetamol and codeine-fixed combination up to 1000 mg and 60 mg, respectively, was administered every 6 hours. In the case of insufficient analgesia as experienced by the patient, oxycodone 1-5 mg iv was given to achieve a NRS<4. If PONV occurred, ondansetron 4 mg iv was administered and followed by droperidol 0.625 mg iv if PONV persisted. Analgesics consumed at home were tramadol and paracetamol/codeine po. Oral morphine equivalents were calculated according to Table 1 (25).

**Table 1:** Equi-analgesic opioid calculations

Medication	Dose	Equi-analgesic ratio
Morphine	1 mg po	1.0
Morphine	1 mg iv	3.0
Oxycodone	1 mg iv	3.0
Oxycodone	1 mg po	1.6
Tramadol	1 mg po	0.1
Codeine	1 mg iv	0.005

*Abbreviations: po – per os; iv – intravenous.

### Outcome measures

The primary outcome was opioid consumption in the first 48 hours postoperatively. In hospital, this information was collected from patients’ medical journals. The consumption at home was self-reported by the patients. The secondary outcomes were self-reported pain at the incision site, deep pain (during coughing), and PONV.

The outcome measures were assessed during the recovery room stay, as well as postoperatively through telephone contact after approximately 24 and 48 hours. The outcome measures were as follows:

• postoperative pain at the incision site and deep pain (when coughing) was self-reported on NRS (where 0 = no pain and 10 = severe pain) 1 and 2 h postoperatively, as well as 5-24 h and 25-48 h postoperatively;

• postoperative pain during mobilization 5-24 h and 25-48 h postoperatively (NRS 0-10);

• opioid analgesic consumption during three time intervals: 0-4 h, 5-24 h, and 25-48 h postoperatively;

• PONV self-reported on a 4-point scale, where 0 = no nausea/vomiting and 3 = severe nausea/vomiting, 0-4 h, 5-24 h, and 25-48 h postoperatively.

### Statistical analysis

Continuous data were tested for normality by the Kolmogorov-Smirnov test. Descriptive data are expressed as mean and standard deviation (SD). The Kruskal-Wallis test was used to test for significant differences between the groups in surgery duration and peroperative dose of propofol/remifentanil, as well as to assess the differences in outcome measures. In addition, the Mann-Whitney U tests were used to compare 1) QLB vs placebo group, 2) QLB vs control group, and 3) placebo vs control group regarding pain, PONV, and opioid consumption. The significance level was set to <0.05. The statistical analysis was performed with the software IBM SPSS Statistics for Windows, version 25.0 (IBM Corp, Armonk, NY, USA) (26).

## Results

In total, 76 patients were recruited. Seventy participants were included: 22 in the control group; 25 in the QLB group, and 23 in the placebo group (Table 2). The groups did not significantly differ regarding surgery duration and propofol/remifentanil dose, and the randomization was successful.

**Table 2:** Patients' demographic data*

	Control group (n = 22)	Quadratus lumborum block group (n = 25)	Placebo group (n = 23)	p†
Sex, n (%)				
male	18 (81.8)	22 (88)	19 (82.6)	0.821
female	4 (18.2)	3 (12)	4 (17.4)	
Age, in years	46.6 (15.9)	45.9 (13.2)	42.7 (13)	0.601
Age range, in years	22-74	22-75	22-69	
Height, cm	170.3 (8.8)	167.2 (7.7)	170.7 (8.8)	0.291
Weight, kg	82.4 (12.9)	79.1 (14.9)	83.3 (16.4)	0.584
Surgery time, min	37.6 (17.9)	35.2 (12.1)	31.1 (14.7)	0.344
Propofol, mg	546.2 (202.6)	480.0 (137.9)	480.4 (239.0)	0.430
Remifentanil, mg	0.73 (0.33)	0.68 (0.28)	0.70 (0.31)	0.366

*Values are presented as mean and standard deviation (SD) unless otherwise indicated.

†Kruskal-Wallis test.

The three groups did not significantly differ in the primary outcome measure. Even though most patients received 5 mg oxycodone peroperatively, 8 of 22 (36%) patients in the control group received approximately 2 mg more than this, compared with 3 of 24 patients (13%) in the QLB group and 6 of 22 patients (27%) in the placebo group (Table 3).

**Table 3:** Postoperative opioid consumption*

	Control group (n = 22)	Quadratus lumborum block group (n = 25)	Placebo group (n = 23)	p^§^
**OME 0-4 h^†^**	17.7 (5.2)	15.4 (7.7)	17.4 (8.9)	0.356
**OME 5-24 h**	12.2 (17.3)	12.3 (12.1)	13.4 (10.1)	0.493
**OME 25-48 h**	14.3 (20.1)	9.2 (6.5)	13.2 (7.7)	0.427
**Tramadol 0-4 h^‡^**	20.5(25.2)	38.0 (43.9)	29.6 (36.7)	0.839
**Oxycodone 0-4 h**	3.3 (5.4)	2.8 (4.1)	3.4 (3.4)	0.166
**Codeine 0-4 h**	4.1 (10.5)	1.3 (6.1)	1.4 (6.4)	0.11
**Tramadol 5-24 h**	100.0 (96.4)	93.8(66.5)	134.1 (79.3)	0.564
**Oxycodone 5-24 h**	2.4 (10.9)	0	0	0.26
**Codeine 5-24 h**	14.3(36.3)	5.0 (16.9)	6.8 (32.0)	0.532
**Tramadol 25-48 h**	56.8 (76.1)	85.0 (82.4)	100.0 (87.3)	0.213
**Codeine 25-48 h**	15.7 (33.7)	5.5 (19.9)	7.1 (32.7)	0.572

*Values are presented as mean and standard deviation.

**†**Primary outcome: equianalgesic dose is expressed in mg morphine per os (OME).

**‡**Dose of oxycodone, codeine, and tramadol in milligrams.

§Kruskal-Wallis test.

Table 4 gives an overview of secondary outcome measures (mean values and standard deviations). If the cut-off for moderate pain was set to NRS = 4-6 and for severe pain to NRS = 7-10 (15,16), 7 of 22 (32%) patients in the control group, 6 of 25 (24%) patients in the QLB group, and 10 of 23 (44%) patients in the placebo group had moderate pain one hour after surgery. Three of 22 (14%) patients in the control group, 3 of 25 (12%) in the QLB group, and 1 of 23 (4%) in the placebo group had severe pain one hour after surgery. A total of 27% of the patients in the control group, 13% of the patients in the QLB group, and 36% of the patients in the placebo group had severe deep pain one hour after surgery (Table 4).

**Table 4:** Secondary outcome measures*

	Control group (n = 22)	Quadratus lumborum block group (n = 25)	Placebo group (n = 23)	*p*^§^
**PONV^†^ 1 h**	0.1 (0.4)	0.2 (0.6)	0.4 (0.7)	0.485
**PONV 2 h**	0.3 (0.7)	0.1 (0.3)	0.4 (0.8)	0.125
**PONV5-24 h**	0.3 (0.7)	0.4 (0.2)	0.2 (0.4)	0.059
**PONV 25-48 h**	0.1 (0.3)	0.1 (0.3)	0.3 (0.7)	0.199
**Pain^‡^ at incision site 1 h**	3.6 (2.3)	3.4 (2.0)	3.6 (2.0)	0.944
**Pain at incision site 2 h**	2.2 (1.2)	2.4 (1.6)	2.9 (1.6)	0.276
**Pain at incision site 5-24 h**	0.3 (2.7)	1.9 (1.5)	2.6 (2.3)	0.317
**Pain at incision site 25-48 h**	2.4 (2.0)	1.5 (1.7)	2.3 (2.0)	0.140
**Deep pain 1 h (cough)**	5.0 (2.5)	4.0 (1.9)	4.8 (2.7)	0.375
**Deep pain 2 h (cough)**	3.3 (1.7)	3.2 (1.5)	4.1 (2.6)	0.243
**Deep pain 4-24 h (cough)**	4.6 (2.0)	3.9 (1.8)	4.4 (2.1)	0.870
**Deep pain 25-48h (cough)**	3.5 (2.0)	3.4 (1.7)	3.9 (2.1)	0.475

*Values are presented as mean and standard deviation.

**†**PONV – postoperative nausea and vomiting, score 0 = none to 3 = severe.

‡Pain score from 0 = none to 10 = severe.

§Kruskal-Wallis test.

The control group and the placebo group did not differ in outcome measures. The control group had a significantly higher PONV score at 2 h (*P* = 0.012) and 3 h (*P* < 0.001), but lower at 5-24 h (*P* = 0.002), and significantly lower pain at incision site at 5-24 h vs the QLB group (Mann-Whitney U, *P* = 0.001). Placebo group had a significantly higher PONV score at 2 h (Mann-Whitney U, *P* = <0.001), 3 h (*P* = 0.001), and 25-48 h (*P* = 0.001), lower PONV at 5-24 h (*P* = 0.001), higher deep pain (cough) at 2 h (*P* = 0.042), and pain at the incision site at 5-24 h (*P* = 0.012) vs the QLB group.

## Discussion

In this study, the groups did not significantly differ in postoperative opioid consumption at any time point. The lack of differences in analgesic consumption and pain scores is in contrast to previous studies comparing the posterior QLB and a sham block with saline in patients undergoing laparoscopic cholecystectomy. One study demonstrated both lower analgesic consumption and lower pain scores in the posterior QLB group (27). Another study showed a significant reduction in postoperative pain with posterior QLB compared with transverse abdominis plane block at all time points (28). Both these studies were conducted without infiltration with local anesthetics around the ports. Moreover, they used intraoperative muscle relaxants, which might have reduced the intraabdominal barotrauma and postoperative pain (29,30). In contrast, we did not use muscle relaxants, but we used local anesthetics for wound infiltration in the ports. The latter have adequate analgesic effects, and are recommended as a part of a multimodal approach for pain relief (31,32). This may be one reason why we did not identify any differences in opioid consumption or postoperative pain.

We only used 20 mL ropivacaine. Cadaver studies have examined the spread of insertion of 30 mL dye solution in the QLB (18,19). Moreover, a study on anterior QLB in cesarean section found beneficial outcomes of inserting 30 mL ropivacaine in the block (33). Hence, increasing the inserted volume of ropivacaine in our study may have altered our results.

Our study did not identify differences between the control, placebo, and the QLB group regarding analgesics consumption or pain score immediately after surgery. These findings are in line with the study by Ishio et al (34), who emphasized that a lack of diffferences may indicate that a sufficient amount of analgesics is administered at the end of the operation. Nevertheless, recent studies showed beneficial analgesic effects of QLB as part of a multimodal approach to reduce postoperative pain following laparoscopic and open surgery (15-17,35). In addition, QLB can be used as rescue analgesia in patients with severe pain and difficulties achieving pain relief (15,16,21).

In the anterior QLB, the needle tip is introduced through the muscle and the anterior fascia. The spread of the injectate is not always easy to visualize, and accidental intramuscular injection of the QL or psoas muscle has been reported (19). Future research on QLB should include cold sensation test or pinprick test to reveal possible block failures.

A recent review of postoperative pain management in laparoscopic cholecystectomy recommended multimodal pain management consisting of paracetamol, NSAIDS, or cyclooxygenase-2 specific inhibitors, and local anesthetic infiltration at the surgical site. Opioids are only recommended for rescue analgesia. Moreover, nerve blocks are not recommended as a routine option, but only when there are no other options for pain management (36). All our patients received multimodal pain management consisting of preoperative paracetamol and NSAIDs, well as peroperative dexamethasone and local infiltration with ropivacaine at the port sites. Hence, it is important that the decision to include QLB is based on the existing evidence that QLB adds benefit to the patients’ recovery.

Clinical experience indicates that patients with insufficient pain relief experience a great effect of nerve blocks such as the anterior QLB. Nevertheless, when placing “rescue-blocks,” patients have already experienced excessive pain, and any relief will result in an experience of effect. Of course, including patients with insufficient pain relief, and providing them with an anterior QLB could have been more beneficial.

We did identify small differences in PONV during 2-48 h postoperatively, with less PONV in the QLB group. The clinical relevance of this finding is unclear.

This study has some limitations. We did not test our patients receiving the block by cold sensation or pinprick test; hence, the QLB may have failed in some patients. On the other hand, the lack of changed sensation in the saline control patients may have biased the study because the patients realized they did not receive local anesthetics. The position of the needle tip relative to the transversalis fascia is of the outmost importance as a slight displacement of the needle tip may lead to a failed block. There were no signs of local anesthetics toxicity, hemodynamic instability or urinary retention, and paralysis of the lower limbs was not observed. Moreover, the ultrasound indicated correct placement and distribution of local analgesics. A further limitation is that the study relied on self-report measures. This may represent a bias in the reporting of opioid consumption after discharge.

Randomized controlled trials comparing outcomes such as pain or patient experiences when using regional nerve blocks vs traditional intravenous and/or oral analgesia in patients undergoing abdominal surgery are not common. Hence, our randomized study adds information about the effects of QLB vs conventional analgesic approaches.

The inclusion of patients from one hospital only, the surgical technique, the anterior placement of the block, as well as the limitations described above may call into question the external validity of our findings. Nevertheless, since we used a sufficient sample size and successfully randomized the patients, the applicability of findings is assumed.

In conclusion, the anterior QLB did not alter postoperative opioid consumption in comparison with placebo block and standard oral/parenteral analgesics following laparoscopic cholecystectomy. Due to uncertain effect of preoperative placement of the QLB, this should not be a routine option, but should be used only when there are no other options for pain management.
